# Ultrasound Evaluation and Grading of Varicoceles: A Two-Cycle Clinical Audit

**DOI:** 10.7759/cureus.93794

**Published:** 2025-10-03

**Authors:** Ibrahim Abuelbeh, Mohammed B Nawaiseh, Hussam Nawaiseh, Rohan Mehra, Qais Nawaiseh, Mohamed Arabiyat, Luke Robinson, Vinotha Nadarajah, Ian Pearce, Vaibhav Modgil

**Affiliations:** 1 Department of Urology, Manchester University National Health Service (NHS) Foundation Trust, Manchester, GBR; 2 Department of Ophthalmology, Jordanian Royal Medical Services, Amman, JOR; 3 Department of Orthopaedic Surgery, Manchester University National Health Service (NHS) Foundation Trust, Manchester, GBR; 4 Department of Medicine, The University of Jordan, Amman, JOR; 5 Department of Urology, Bolton National Health Service (NHS) Foundation Trust, Bolton, GBR; 6 Department of Radiology, Manchester University National Health Service (NHS) Foundation Trust, Manchester, GBR

**Keywords:** clinical audit, esur-spiwg guidelines, imaging guidelines, infertility, scrotal imaging, structured reporting, ultrasound, varicocele

## Abstract

Introduction

Varicoceles are a common cause of male infertility, and ultrasound (US) is the primary imaging modality used for diagnosis and grading. However, variability in reporting practices can hinder clinical decision-making. This clinical audit aimed to improve adherence to standardized US reporting guidelines for varicoceles, as recommended by the European Society of Urogenital Radiology Scrotal and Penile Imaging Working Group (ESUR-SPIWG) and the Royal College of Radiologists (RCR).

Materials and methods

A two-cycle retrospective clinical audit was conducted in Manchester University NHS Foundation Trust. Testicular US reports were assessed before and after a targeted intervention that included a multidisciplinary meeting and the implementation of a structured reporting template. Reports were evaluated against ESUR-SPIWG and RCR guidelines, focusing on documentation of testicular volume, varicocele location, largest vein diameter, reflux assessment, and varicocele grading using the Sarteschi classification. Statistical analysis was performed using the chi-square test.

Results

A total of 66 testicular US reports were reviewed across two audit cycles. Statistically significant improvements were observed post-intervention in the documentation of key parameters (all p < 0.001): testicular volume (0.0% to 91.6%), varicocele location (0% to 91.6%), largest vein diameter (50.0% to 100%), and Valsalva/varicocele grading (0% to 97.2%).

Conclusions

The introduction of a structured reporting template and interdisciplinary collaboration significantly improved compliance with international varicocele US reporting standards. This approach enhances diagnostic quality and supports more effective urological triage. The findings suggest a scalable model for broader adoption and future integration into electronic systems, promoting consistency, auditability, and improved patient outcomes.

## Introduction

Varicoceles are a common urological condition affecting approximately 15% of the male population and constitute the leading correctable cause of male infertility [1]. They result from abnormal dilatation of the pampiniform venous plexus, often impairing testicular function and semen parameters [2]. While many varicoceles are asymptomatic, their presence has been linked to infertility, testicular atrophy, and scrotal pain [3,4].

Ultrasound (US) remains the first-line imaging modality for evaluating varicoceles due to its accessibility, non-invasiveness, and ability to assess venous reflux dynamically [5]. The Sarteschi classification system, endorsed by the European Society of Urogenital Radiology Scrotal and Penile Imaging Working Group (ESUR-SPIWG), is increasingly recognized as the preferred grading system due to its comprehensive, position-sensitive assessment of reflux across three anatomical locations: inguinal, supratesticular, and peritesticular [5,6]. Notably, reflux lasting more than two seconds during a Valsalva maneuver is strongly associated with improved postoperative semen quality, highlighting the prognostic significance of accurate reflux assessment [5].

In an effort to harmonize clinical practice, the ESUR-SPIWG has issued evidence-based guidelines for standardizing varicocele evaluation using US [7]. These guidelines recommend that all reports should include testicular volume, measured using Lambert's formula (length × width × height × 0.71); location; diameter of the largest vein in both supine and erect positions; and presence and anatomical level of venous reflux in both positions (inguinal, supratesticular, peritesticular) [7].

In the context of improving current practice within our institute, Manchester University NHS Foundation Trust, this project aimed to introduce a targeted intervention to align sonographic reporting with the ESUR-SPIWG guidelines. By standardizing reporting parameters, the project aimed to enhance the diagnostic consistency of varicocele evaluations, thereby streamlining urological triage and optimizing patient management and outcomes.

## Materials and methods

This audit was conducted at our institute to align varicocele US reporting with the standardized criteria recommended by the ESUR-SPIWG and the Royal College of Radiologists (RCR) recommendations. The project employed a retrospective audit methodology consisting of two audit cycles conducted between January and October 2024. First-cycle data were collected from January 10, 2024, to February 24, 2024. The intervention lasted from March to July 2024, and second-cycle data were collected from July to September 2024. The audit was finalized and presented in October 2024. The sample was consecutive and included every US report that had a finding of varicocele.

Data for both audit cycles were collected retrospectively through the Trust’s electronic patient system. Inclusion criteria comprised all patients who underwent scrotal US and were found to have varicoceles. The first audit cycle consisted of 30 cases, while the second audit cycle comprised 36 cases. These samples were chosen based on the availability and completeness of the US reports during the respective periods. Several sonographers and radiologists, both from the same Trust and locum practitioners from external agencies, reported US scans. The same reviewer conducted the data collection, and a second reviewer independently verified it to enhance consistency and minimize potential bias.

The standard against which reports were evaluated was derived directly from the ESUR-SPIWG guidelines and the RCR recommendations, along with an agreement between urology and radiology departments within the Trust, which includes measurement of testicular volume using Lambert’s formula (length × width × height × 0.71), identification of the anatomical location of the varicocele (inguinal, supratesticular, or peritesticular), documentation of the diameter of the largest vein, assessment of venous reflux using a Valsalva maneuver, along with grading of the varicocele using the Sarteschi classification [5,8].

Each US report was reviewed to determine compliance with the agreed criteria. Data collection was performed manually and recorded using a standardized proforma, which was created following a multidisciplinary meeting involving a consultant urologist, a consultant radiologist, and the lead sonographer. The proforma was developed based on evidence-based guidelines, and input from the three representatives was incorporated to ensure a balance between guideline adherence, radiologist and sonographer preferences, and the practical requirements of the urology service.

Following the first audit cycle, a targeted intervention was implemented to improve adherence to the recommended reporting standards. This intervention had two key components. First, the findings from the initial audit were presented at a multidisciplinary meeting attended by representatives from both the radiology and urology departments. The objective of this meeting was to raise awareness of the reporting guidelines and to emphasize the clinical relevance of comprehensive reporting for urological triage and surgical decision-making. Second, a dedicated sonographic reporting template was developed and introduced (see the Appendices section). This template was designed in collaboration between urologists and radiologists to ensure usability and clinical relevance. It included structured fields for all core reporting parameters outlined in the guidelines. The reporting template was based on the ESUR-SPIWG guidelines, and the varicocele grading used was the Sarteschi grading system [5-7,9]. All tools used in this article are free to use.

The second audit cycle was conducted several months after the intervention's implementation, using the same methodology and inclusion criteria as the initial cycle. The aim was to assess the impact of the intervention on the quality and completeness of reporting.

Data were analyzed using SPSS Statistics version 30 (IBM Corp. Released 2024. IBM SPSS Statistics for Windows, Version 30.0. Armonk, NY: IBM Corp.). Descriptive statistics were used to describe categorical variables in terms of frequencies and percentages. Comparisons between the first and second audit cycles were performed using the chi-square test to assess improvements in the documentation of key US reporting parameters, including testicular volume, varicocele location, largest vein diameter, and Valsalva/varicocele grading. A p-value of <0.05 was considered statistically significant.

No patient-identifiable information was collected or analyzed during this project. Prior to accessing patient records, approval was obtained from the Clinical Audit and Effectiveness Department at our institute to ensure compliance with ethical and governance standards. All data were handled in accordance with Trust policies on confidentiality and data protection.

## Results

A total of 66 testicular US reports were reviewed, comprising 30 scans from the first audit cycle (pre-intervention) and 36 scans from the second cycle (post-intervention). Substantial improvements in documentation were observed following the intervention. Testicular volume, which was not recorded in any of the first-cycle reports (0.0%), was documented in 33 of 36 second-cycle reports (91.6%), with a Pearson chi-square value of 44.892 (p < 0.001). The location of varicocele, absent in all first-cycle reports, was recorded in 33 second-cycle reports (91.6%), with a Pearson chi-square of 48.714 (p < 0.001). Documentation of the largest vein diameter increased from 15 first-cycle reports (50.0%) to all second-cycle reports (100.0%), Pearson chi-square = 23.294 (p < 0.001). The use of the Valsalva maneuver and grading of varicoceles improved from 0.0% in the first cycle to 35 of 36 reports (97.2%) in the second cycle, with a Pearson chi-square of 66.000 (p < 0.001). Overall, the post-intervention cycle demonstrated consistent adherence to recommended reporting standards, with statistically significant improvements across all measured parameters (see Table 1).

**Table 1:** Comparison of key US reporting parameters between first and second audit cycles in testicular varicocele assessment * chi-square test, US: ultrasound

Variable	First cycle	Second cycle	Pearson chi-square	p-value*
Number	30	36		
Testicular volume	0 (0.0%)	33 (91.6%)	44.892	<0.001
Location	0 (0.0%)	33 (91.6%)	48.714	<0.001
Largest vein diameter	15 (50.0%)	36 (100.0%)	23.294	<0.001
Valsalva and varicocele grading [5,9]	0 (0.0%)	35 (97.2%)	66.000	<0.001

## Discussion

Varicoceles affect roughly 15% of adult men and represent the most common surgically correctable cause of male infertility [5]. As a result, accurate detection and characterization of varicoceles are crucial for fertility counseling and treatment planning [5,10]. US is the modality of choice for varicocele assessment [5]. It provides objective measurements of venous anatomy and reflux, overcoming the limitations of physical examination, such as the detection of subclinical varicoceles, which can only be done through imaging [11]. Under standardized US protocols, in both supine and standing positions with and without Valsalva, dilated pampiniform veins appear as tubular anechoic structures that enlarge with Valsalva [5,11]. Quantitative findings on US have direct prognostic implications: a venous diameter of ≥3 mm and reflux lasting for >2 seconds during Valsalva are widely accepted criteria for a varicocele [12-15]. Moreover, graded Doppler findings such as Sarteschi grading correlate with outcomes. In particular, persistent reflux of more than two seconds has been shown to predict postoperative improvement in semen parameters, and ipsilateral testicular atrophy and reduced volume are common in significant varicoceles and often reverse after repair [2,5,16]. Thus, a comprehensive US evaluation, including testicular volumes and reflux grading, directly influences urologists’ decisions regarding varicocele.

To ensure that all clinically significant findings are documented, international guidelines recommend a structured approach to varicocele US. The ESUR-SPIWG guidelines recommend a standardized examination and report, specifying key data fields [5]. For example, reports should include testicular volumes measured by Lambert’s formula and the location and diameter of the largest vein in both supine and erect positions. Crucially, reports should note the level and duration of venous reflux, either inguinal, supravesical, or perivesical, with Valsalva in each position, and the varicocele grade, preferably Sarteschi’s classification. These criteria were also recommended by RCR and are designed to eliminate variability [8]. Structured reporting ensures completeness and comparability of reports by providing a built-in checklist [17]. In our audit, a multidisciplinary-developed template embedded these fields directly into the report. The template ensured a balance between important details required by urologists to aid in diagnosis and management and the sonographer and radiologist's limited time slots for the US. Such templates implement the guidelines, functioning as helpful aids for sonographers and radiologists. Indeed, structured formats and standardized terminology have been shown to prevent omissions and ambiguity, thereby improving the accuracy and utility of radiology reports [17].

Multiple previous audits were done to help standardize reporting. For example, a recent closed-loop audit was conducted to assess soft-tissue US reporting and compliance with RCR reporting criteria, which increased from 41.5% to 98.3% after the implementation of structured reporting templates and feedback [18]. In that study, it was concluded that integrating methods such as structured report templates can significantly increase adherence to guidelines and enhance the comprehensiveness and reliability of reports [18]. Our two-cycle audit demonstrated that raising awareness and implementing a structured reporting system dramatically increased compliance with these standards, as documentation of all key parameters improved significantly from near-zero levels at baseline to over 90% post-intervention. Our findings corroborate this effect in the context of scrotal imaging. By explicitly citing the clinical rationale at the multidisciplinary meeting and then providing a reporting template, the audit managed to make a difference. The combined measures of education, along with practical tools, were markedly more effective than either measure alone. No prior internal data were available for comparison; however, the magnitude of improvement and statistical significance indicate that the intervention, rather than secular trends, drove the change.

Standardized US reporting has significant benefits. First, consistency enables meaningful auditing and benchmarking across centers. If all trusts adopt the same ESUR-SPIWG and RCR reporting standards, it becomes feasible to compare compliance rates and patient outcomes on a national level. Standardization was needed not only to improve clinical practice but also to define the methodological basis for future studies and meta-analyses [5]. By reporting structured data, centers can pool information for research or registries, facilitating cross-site quality comparisons and assessments. Second, standardized reports improve clinical triage and surgical planning. Urologists rely on US data to stratify patients; for instance, significant asymmetry in testicular volume or high-grade bilateral reflux may prioritize a man for intervention. Conversely, a trivial asymptomatic varicocele with normal volumes might be managed conservatively. Clear documentation of location also alerts surgeons to potential atypical anatomy. Consistent terminology minimizes misinterpretation, a feature emphasized by proponents of structured reports [17]. Finally, comprehensive reports ensure that no key feature is overlooked when deciding on surgery. For example, noting reflux in the contralateral testicular veins, often subclinical, can prompt bilateral exploration if indicated. In short, by capturing all relevant findings uniformly, standardized US reports directly support timely referral, operative planning, and benchmarking of outcomes.

This audit was conducted at a single tertiary center and its affiliated Trust hospitals, with a modest case number of 66 studies, which may limit generalizability. Additionally, as part of the service evaluation, we did not collect patient outcome data, such as surgery rates or fertility results; therefore, the audit focuses solely on process measures. Finally, not all sonographers and radiologists who report the scans work in the center, as they might be locum shift workers or employed by different companies, which makes intervention more challenging. Nonetheless, the magnitude of change and statistical robustness suggest an actual effect of the intervention rather than chance or seasonal variation. Additionally, the audit findings were presented at regional and national meetings in urology, radiology, and sonography. Additionally, the reporting template was added to the sonographer induction handbook, which helped promote widespread adherence to the guidelines, regardless of the sonographer's original work location.

An often underemphasized yet critical element in clinical audits and quality improvement projects is the collaboration between different specialties. The success of this audit hinged not only on technical implementation but also on the active partnership between the urology and radiology teams. Multidisciplinary engagement ensured that the template was both clinically relevant and practically usable, aligning with broader healthcare literature that emphasizes the importance of involving end-users in the design and rollout of interventions for sustainable improvements in diagnostic accuracy and workflow efficiency [19]. This collaborative model could serve as a blueprint for other imaging domains where the clinical utility of radiology reporting depends on shared understanding between referring and reporting clinicians.

The encouraging results suggest that similar audit cycles should be adopted more widely. We plan to share our report template and methodology nationwide in the United Kingdom, utilizing the same ESUR-SPIWG and RCR criteria, which could standardize varicocele care and facilitate cross-institutional benchmarking. Future work might include extending the post-intervention cycle to assess long-term adherence, as well as linking report quality to surgical and fertility outcomes. The integration of the template into the electronic reporting system could sustain these improvements. Ultimately, a national quality improvement initiative could ensure that all men with varicoceles receive high-quality sonographic assessment, optimizing triage and treatment decisions.

## Conclusions

This audit demonstrates that the implementation of structured US reporting templates, combined with multidisciplinary engagement, results in significant improvements in the quality and consistency of US varicocele assessments. Following the intervention, compliance with the agreed-upon guidelines increased significantly across all key reporting parameters.

These findings support the integration of standardized protocols in routine clinical practice to enhance diagnostic reliability and facilitate more effective urological triage. The structured format also enables ongoing quality assurance through audit and benchmarking. While further evaluation across broader clinical settings is warranted, this approach offers a scalable model for improving imaging quality in varicocele assessment and beyond.

## Appendices

**Table 2:** US reporting template US: ultrasound

Category	Details
Testicular volume	Right *** cc, left *** cc
Varicocele side	Right/left/bilateral
Varicocele location	Inguinal/supra-testicular/peri-testicular
Largest vein diameter	*** mm
Valsalva maneuver	Performed/not performed
Varicocele grading [5,9]	Grade I - No dilated intra-scrotal veins. Reflux in spermatic cord veins of the inguinal region during the Valsalva maneuver. Grade II - Prominent veins at the upper pole of the testis (pampiniform plexus). Reflux during the Valsalva maneuver. Grade III – Dilated veins only when standing. Reflux during the Valsalva maneuver. Grade IV - Dilated veins even in the supine position. Reflux during the Valsalva maneuver. Grade V - Dilated veins, Reflux without the Valsalva maneuver. Testicular hypotrophy
Kidneys scanned?	Yes/no, if not, please state why?
